# HIV-1 genetic diversity and resistance to antiretroviral drugs among pregnant women in Ribeirão Preto (SP), Brazil. Cross-sectional study

**DOI:** 10.1590/1516-3180.2017.0233011017

**Published:** 2018-01-09

**Authors:** Ana Teresa Mancini Pimenta, Isadora Alonso Correa, Patricia Pereira dos Santos Melli, Renata Abduch, Geraldo Duarte, José Carlos Couto-Fernandez, Silvana Maria Quintana

**Affiliations:** I MSc, PhD. Biologist, Department of Gynecology and Obstetrics, Faculdade de Medicina de Ribeirão Preto (FMRP), Universidade de São Paulo (USP), Ribeirão Preto (SP), Brazil; II BSc. Biologist, Fundação Oswaldo Cruz (Fiocruz), Rio de Janeiro (RJ), Brazil; III MD, PhD. Attending Physician, Department of Obstetrics and Gynecology, University Hospital, Faculdade de Medicina de Ribeirão Preto (FMRP), Universidade de São Paulo (USP), Ribeirão Preto (SP), Brazil; IV MD. Physician, Department of Obstetrics and Gynecology, Faculdade de Medicina de Ribeirão Preto (FMRP), Universidade de São Paulo (USP), Ribeirão Preto (SP), Brazil; V MD, PhD. Professor, Department of Obstetrics and Gynecology, Faculdade de Medicina de Ribeirão Preto (FMRP), Universidade de São Paulo (USP), Ribeirão Preto (SP), Brazil; VI MSc, PhD. Researcher, Laboratory of AIDS and Molecular Immunology, Fundação Oswaldo Cruz (Fiocruz), Rio de Janeiro (RJ), Brazil; VII MD, PhD. Associate Professor, Department of Obstetrics and Gynecology, Faculdade de Medicina de Ribeirão Preto (FMRP), Universidade de São Paulo (USP), Ribeirão Preto (SP), Brazil

**Keywords:** HIV-1, Pregnancy, Drug resistance

## Abstract

**BACKGROUND:**

Increasing genetic diversity of HIV-1 and emergence of drug-resistant mutations may reduce the efficacy of antiretroviral therapy and prophylaxis that are used to prevent mother-to-child transmission. The aim of this study was to assess the genetic diversity and prevalence of drug-resistant mutations among HIV-infected pregnant women.

**DESIGN AND SETTING:**

Cross-sectional study at an outpatient clinic for infectious diseases within gynecology and obstetrics.

**METHODS:**

This study evaluated the dynamics of HIV-1 subtypes and the prevalence of transmitted and acquired drug-resistant mutations among 38 HIV-infected pregnant women (20 previously exposed to antiretroviral therapy and 18 naive), in Ribeirão Preto (SP), Brazil, between 2010 and 2011. Genotyping was performed by means of molecular sequencing of the protease and reverse transcriptase regions of the HIV-1 pol gene.

**RESULTS:**

Subtype B was identified in 84.2% of the samples, recombinant forms between B and F in 7.9%, subtype F1 in 5.3% and the recombinant form K/F in 2.6%. No mutation associated with transmitted drug resistance was detected in the samples from the naive pregnant women, whereas mutations associated with acquired drug resistance were found in 35.0% of the pregnant women previously exposed to antiretroviral therapy.

**CONCLUSION:**

The results showed that subtype B predominated, while there was low prevalence of sequences with transmitted drug resistance.

## INTRODUCTION

The estimates show that 781,000 people in Brazil are infected with the human immunodeficiency virus (HIV-1), of whom 35.0% are women.^1^ In 2015 there were 7,901 HIV-infected pregnant women, with an estimated detection rate of 2.7 per 1,000 live births.^2^ In the state of São Paulo alone, in 2014, it was estimated that there were 2,616 HIV-infected pregnant women.^3^

Preventive interventions such as combined antiretroviral therapy (cARVT), delivery by means of caesarean section, chemoprophylaxis using zidovudine (for both parturient and newborn) and avoidance of breastfeeding have decreased HIV-1 mother-to-child transmission throughout the world, to levels below 2.0% in some regions.^4^ The Brazilian government has provided free access to cARVT to all HIV-1-infected individuals through the National Health System (Sistema Único de Saúde, SUS)^5^ since 1996 (zidovudine distribution started in 1991).^6^ These actions have resulted in stabilization of disease prevalence over the last few years,^1^ decreases in HIV/AIDS-related mortality and morbidity,^7^ and reduction in mother-to-child transmission to 3.4 per 100,000 inhabitants under 5 years of age in 2012.^8^ However, selection of HIV-1 resistant variants during treatment or transmission significantly impacts the effectiveness of cARVT, thus compromising the sustainability of national treatment, care and prevention programs.

Antiretroviral resistance arises from mutations in the viral genes that encode the molecular targets of therapy.^9^ Although the prevalence of transmitted drug resistance is still low in Brazil, it has increased significantly, especially in the Southeast, the most populous region of the country.^10^^,^^11^ This reinforces the need for epidemiological studies on groups that are vulnerable to infection in Brazil, in particular in regions of the country that are neither state capitals (or the national capital) nor coastal regions.^1^ The aim of this study was to assess the genetic diversity and prevalence of drug-resistant mutations among HIV-infected pregnant women.

## METHODS

### Ethical considerations and study population

The project and informed consent statement were approved both by the Research Committee of the Department of Gynecology and Obstetrics and by the Ethics Committee of the University Hospital of Ribeirão Preto School of Medicine, University of São Paulo (procedural no. 13411/2009). All participants were informed about the aim of the study and signed the consent statement before participation.

This study included 38 HIV-1-infected pregnant women who were living in Ribeirão Preto, São Paulo, Brazil. They were enrolled during prenatal care at the outpatient clinic for infectious diseases within gynecology and obstetrics of the University Hospital, Ribeirão Preto School of Medicine, University of São Paulo, between March 2010 and September 2011. Blood samples were collected before the patients started to receive cARVT or before any treatment switch, and plasma was stored in a freezer at -70 °C. Data on the diagnoses and the clinical and epidemiological characteristics of the pregnant women, their use of cARVT during prenatal care and their antiretroviral regimens were obtained from the women’s medical records.

### Gene sequencing

Viral RNA was extracted by using the QIAamp Viral RNA minikit (QIAGEN, Germany), with reverse transcription into cDNA (RT-PCR) and polymerase chain reaction (PCR) amplification using both “in-house” methodology and the Trugene commercial system (Trugene HIV-1 genotyping assay, Siemens Diagnostics, USA). The entire protease (PR) and 75% of the reverse transcriptase (RT) were sequenced in an automatic sequencer. All DNA sequences were analyzed using the OpenGene DNA sequencing system (Trugene, Siemens) and, for the ‘in-house’ sequencing, the Applied Biosystems ABI3130xl system (Applied Biosystems, USA). Chromatograms were verified using the SeqMan v7 software (LaserGene; DNAStar, USA) and were then compared with HIV-1 reference sequences. The sequences obtained here were submitted to GenBank under the accession numbers MF554758 to MF554780 and MF669076 to MF669087.

### Resistance and phylogenetic analysis

To define the HIV-1 genetic subtype, interpretations of DNA sequences were made using both the Brazilian algorithm and the REGA HIV-1 tool, version 3.0. The transmitted drug resistance profile was evaluated using the Stanford HIV resistance database, and presence of transmitted drug resistance was evaluated using the Stanford Calibration of Resistant Population (CRP) tool [Stanford HIV-1 Drug Resistance Mutation (SHDRM) database]. Recombinant samples were evaluated by means of the simplot and bootscanning software, which compares sequences with reference sequences in the Los Alamos HIV database. Phylogenetic trees were constructed by aligning the sequences with the most representative reference sequences of the various pure and recombinant subtypes of the HIV-1 M group, which were obtained from the Los Alamos HIV-1 database. Maximum-likelihood (ML) trees were estimated using the MEGA 6 software. Bootstraps of 1,000 replicates were used and values greater than 70% were presented in the trees.

A significance level of 5% was used for all statistical tests. These tests were performed in the SAS software, version 9 (Statistical Analysis System, SAS Institute Inc, USA).

## RESULTS

### Characteristics of the study population

Eighteen pregnant women (47.4%) were naive, while 20 (52.6%) had already used antiretroviral therapy before the current pregnancy or were using it during the pregnancy (cARVT-exposed). Most of the women enrolled (81.6%) had been diagnosed with HIV infection during prenatal care for the current or a previous pregnancy.

**Table 1** presents the characteristics of the pregnant women included. The mean age of the naive pregnant women was 26.4 years (standard deviation, SD = 6.0), while the mean age of the cARVT-exposed pregnant women was 29.2 years (SD = 4.0). The mean numbers of pregnancies and deliveries were higher among the cARVT-exposed women: on average, these women began prenatal care at an earlier gestational stage than did the naive pregnant women (35.0% vs. 21.0% in the first trimester). Most participants (86.8%) were asymptomatic. Viral load distribution was similar between the cARVT-exposed and naive pregnant women (P = 0.65), although with greater variation in the cARVT-exposed women. The median CD4+ T lymphocyte count was greater among the cARVT-exposed pregnant women, and the median CD8+ T lymphocyte count was greater among the naive pregnant women, but there was no statistical difference for either of these parameters between the two groups (P = 0.76 and P = 0.59, respectively).

**Table 1.** Characteristics of HIV-1 infected pregnant women stratified according to combined antiretroviral therapy (cARVT) use. Ribeirão Preto (SP), Brazil, 2010-2011

Variables	Naive (n = 18)	cARVT-exposed (n =20)
**Age, years, mean (SD)**	26.4 (6.0)	29.2 (4.0)
**Race, n (%)**		
Black	2 (11.1)	2 (10.0)
Mixed race	5 (27.8)	5 (25.0)
White	11 (61.1)	14 (70.0)
**Education, n (%)**		
≤ 8 years	14 (77.8)	16 (80.0)
9-11 years	4 (22.2)	4 (20.0)
**Marital status, n (%)**		
Cohabitating	7 (38.9)	9 (45.0)
Divorced/separated	2 (11.1)	2 (10.0)
Married	5 (27.8)	4 (20.0)
Single	4 (22.2)	5 (25.0)
**Illicit drug use, n (%)**	8 (44.4)	6 (30.0)
**Start of prenatal care, mean (SD)**	22 w + 6 d (10 w + 6 d)	17 w + 6 d (7 w)
**Number of pregnancies, mean (SD)**	3.7 (2.4)	4.7 (2.5)
**Number of deliveries, mean (SD)**	2.2 (2.4)	3.2 (2.0)
**Viral load, copies/ml, median (IQ)**	9,521 (3,847–27,985)	10,995 (4,977–43,110)
**CD4+ T lymphocytes, cells/mm^3^, median (IQ)**	299 (193–554)	333 (270–392)
**CD8+ T lymphocytes, cells/mm^3^, median (IQ)**	812 (624–947)	694 (535–950)

SD = standard deviation; w = weeks; d = days; IQ = interquartile range.

At the first outpatient visit, it was observed that, among the 20 cARVT-exposed pregnant women, six were undergoing a therapy regimen. In this subgroup, 27.8% had used or were using monotherapy, whereas 15.0% had used a three-drug regimen. The most commonly used drug combination was zidovudine + lamivudine + nelfinavir (50.0%), followed by zidovudine + lamivudine + lopinavir/ritonavir (44.4%), zidovudine + lamivudine + efavirenz (22.2%) and zidovudine + lamivudine + other drugs (11.1%). Because the drug combinations used varied over time, the percentages do not total 100%.

In addition to HIV infection, 38.9% of the naive and 50.0% of the cARVT-exposed pregnant women presented other sexually transmitted infections (STIs), like syphilis, trichomoniasis, *Ureaplasma*, genital warts or grade I cervical intraepithelial neoplasia. STIs plus genital infection (vaginal candidiasis and bacterial vaginosis) were also observed in 22.2% of the naive pregnant women and in 10.0% of the cARVT-exposed pregnant women. There were no statistically significant differences in viral load or in CD4+ and CD8+ T lymphocyte counts among women with STIs (P = 0.93, P = 0.41 and P = 0.26, respectively) or among those with STIs plus genital infections (P = 0.23, P = 0.14 and P = 0.18, respectively), in comparison with the women who did not have any of these conditions.

### Diversity of HIV subtypes

Subtype B was identified in 84.2% of the samples, F1 in 5.3%, recombinant forms B/F and F/B in 7.9% and the unique recombinant form (URF) K/F in 2.6%. Most circulating recombinant forms (CRFs) were composed of subtypes B and F1, and phylogenetic analysis showed that these had a close relationship to CRF29_BF, which was described in the city of São Paulo (SP), Brazil (http://www.hiv.lanl.gov/content/sequence/HIV/CRFs/CRFs.html). Among the naive pregnant women, 83.4% presented subtype B and 5.6% had subtype F1, while the recombinant forms B/F and K/F were also present in one pregnant woman each. Among the cARVT-exposed pregnant women, subtype B was also the most prevalent, with an incidence of 85.0%, followed by the recombinant form B/F (10.0%) and subtype F1 (5.0%).

**Figure 1.**
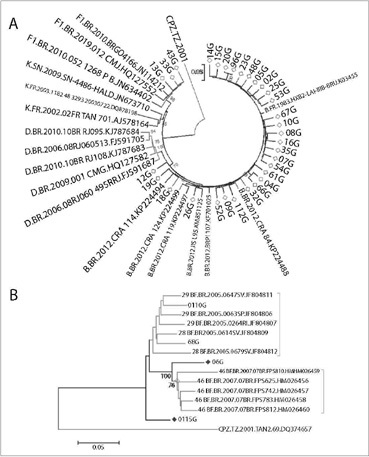
Subtype classification of sequences. A) Maximum likelihood tree of PR/RT region. HIV-1 reference sequences of pure subtypes (B, D, F1 and K) were included. The branch support values (aLRT > 0.90) are indicated at key nodes. Each HIV-1 clade found in the Brazilian samples is indicated in the figure. Horizontal branch lengths are drawn to scale with the bar at the bottom indicating nucleotide substitutions per site. B) ML tree of PR/RT region of the inter-subtype recombinant samples. HIV-1 reference sequences of CRFs.

Subtype F1 sequences comprised a separate cluster, and were identical to sequences from the southeastern region of Brazil. A cluster among subtype B sequences was observed, suggesting that there was an epidemiological relationship between the different viral isolates and, consequently, that viruses were introduced into the region in a monophyletic fashion with subsequent spreading of these subtypes in the region. Identification of highly divergent recombinant samples (115) and other recombinants (68 and 110) suggested that these viruses have only recently been introduced among pregnant women in Ribeirão Preto (**Figure 1**).

### Prevalence and drug resistance patterns of HIV

No major mutation responsible for drug resistance was present in the sequences obtained from the naive pregnant women. However, three samples (16.7%) had accessory mutations to non-analogous nucleoside reverse transcriptase inhibitors (NNRTIs): mutations V108I, E138A and V179D, associated with efavirenz and rilpivirine (**Table 2**). Accessory mutations related to protease inhibitors (PIs), such as the L10I and K20R polymorphisms, were also detected in one sample (5.6%). No case of mutation associated with analogous nucleoside/nucleotide reverse transcriptase inhibitors (NRTI) was observed.

**Table 2.** HIV-1 subtypes and resistance mutations associated with analogous nucleoside/nucleotide reverse transcriptase inhibitors (NRTI), non-analogous nucleoside reverse transcriptase inhibitors (NNRTI) and protease inhibitors (PI)

	PW*	Subtype	Drug-resistant mutations			Other mutations		
			NRTI	NNRTI	PI	NRTI	NNRTI	PI
**Naive**	04G	B	-	-	-	-	-	-
	06G	B/F	-	-	-	-	-	-
	09G	B	-	-	-	-	V108I	-
	13G	K/F	-	-	-	-	-	-
	14G	B	-	-	-	-	-	-
	18G	B	-	-	-	-	-	-
	19G	B	-	-	-	-	-	-
	23G	B	-	-	-	-	-	-
	25G	B	-	-	-	-	V179D	-
	26G	B	-	-	-	-	-	-
	33G	F	-	-	-	-	-	-
	48G	B	-	-	-	-	-	-
	52G	B	-	-	-	-	E138A	-
	54G	B	-	-	-	-	-	-
	68G	B	-	-	-	-	-	L10I, K20R
	84G	B	-	-	-	-	-	-
	95G	B	-	-	-	-	-	-
	112G	B	-	-	-	-	-	-
**Exposed to cARVT**	2G	B	M184V	-	-	-	-	-
	05G	B	-	-	-	-	-	-
	07G	B	D67N, T69D, K219R	-	-	-	-	-
	08G	B	-	-	-	-	-	-
	10G	B	M184V	-	-	-	P225H	-
	12G	B	-	-	-	-	-	-
	15G	B	-	-	-	-	-	-
	16G	F/B	-	-	-	-	-	L10V, K20R
	20G	B	-	-	M46IL	-	-	-
	32G	B	-	-	-	-	-	-
	35G	B	-	-	-	-	-	-
	43G	F	-	-	-	-	-	-
	53G	B	-	-	-	-	-	-
	61G	B	-	-	-	-	E138A	-
	66G	B	-	-	-	-	-	-
	67G	B	-	-	-	-	-	-
	96G	B	-	-	M46L	-	-	-
	98G	B	D67N, T69N, K70R, M184V, T215F, K219EQ	-	I50L	-	-	-
	110G	F/B	-	K103N	-	V75M	-	-
	115G	B	-	-	-	-	-	L10V, K20R

cARVT = combined antiretroviral therapy; PW = pregnant women; NRTI = nucleoside/nucleotide reverse transcriptase inhibitors; NNRTI = non-analogous nucleoside reverse transcriptase inhibitors.

Among the sequences from the cARVT-exposed pregnant women, 35.0% displayed resistance to antiretroviral drugs. Four sequences (20.0%) presented drug-resistant mutations to NRTI, 15.0% to PIs and 5.0% to NNRTIs. The M184V mutation was the most prevalent (15.0%), followed by mutations associated with RT thymidine inhibitors (TAM) (10.0%). The V75M mutation associated with resistance to didanosine and stavudine was detected in one sample (5.0%). Three samples displayed K103N, E138A and P225H mutations, one per sample. Two samples showed concomitant mutations associated with NRTI and PI (sample 98), and NRTI and NNRTI (sample 110). Two samples had the M46L mutation (10.0%) and one had the I50L mutation (5.0%) associated with atazanavir. Also, the most prevalent accessory mutations were L10I/V and K20M/R (10%), which were detected in two sequences (samples 16 and 115). None of the samples from the cARVT-exposed pregnant women showed resistance to three classes of drugs. The number of antiretroviral regimens previously used was associated with the presence of drug-resistant viruses (P = 0.041).

The cARVT-exposed pregnant women had 2.10-fold higher prevalence of antiretroviral resistance (95% CI = 0.64-6.93), compared with the naive pregnant women, and 2.03-fold greater drug resistance and intermediate drug resistance (95% CI = 0.75-5.45). The prevalence of antiretroviral drug resistance was 11.2% in the samples from the naive pregnant women with intermediate resistance to etravirine, whereas only 5.0% of the samples from the cARVT-exposed pregnant women showed possible resistance to the same antiretroviral drug. Possible resistance to didanosine was the most prevalent form of resistance among the cARVT-exposed pregnant women (20.0%), followed by resistance to lamivudine (15.0%). Resistance to efavirenz was observed in 10.0% of the cARVT-exposed pregnant women. Resistance to fosamprenavir and to indinavir was also observed in 10.0% of the cARVT-exposed pregnant women.

Polymorphisms in protease genes were observed in all the samples from the naive pregnant women, with the exception of one sample, and in three samples from the cARVT-exposed pregnant women. The polymorphism M36I/M36L was the most frequent, present in 12 samples from the naive pregnant women (66.7%) infected with HIV-1 subtypes B, F, B/F and K/F, and in 10 samples (50.0%) from the cARVT-exposed pregnant women infected with HIV-1 subtypes B, F and B/F. Next came L63P/L63Q/L63S, present in 33.3% of the naive pregnant women and in 20.0% of the cARVT-exposed pregnant women; and L10I/L10V in 22.2% of the naive pregnant women and in 25.0% of the cARVT-exposed pregnant women. The M36I mutation was not associated with any virus subtype (P = 0.77).

No case of mother-to-child transmission of HIV was diagnosed among the pregnant women included in this study.

## DISCUSSION

In this study, subtype B was the most common form of HIV-1 (84.2%) found among the pregnant women in Ribeirão Preto. This was consistent with other data from Brazil^12^^,^^13^^,^^14^^,^^15^^,^^16^^,^^17^^,^^18^^,^^19^^,^^20^ with the exception of the southern states, where subtype C has been found to prevail.^21^ To a lesser extent, presence of recombinant forms between subtypes B and F, K and between F and subtype F1 was also found. Subtype C was not observed in the samples, but the prevalence of this subtype is increasing in Brazil,^22^ apparently with disease progression characteristics differing from those of subtype B.^23^ Subtype F is the second most common form in the cities of Santos^24^^,^^25^ and Rio de Janeiro^18^ and in the states of Minas Gerais^19^ and Pará.^20^ Despite the small sample size, the number of samples presenting recombinant subtype B/F and two highly divergent recombinant samples because of genetic diversity and the complexity of HIV epidemic dynamics was striking. Subtype F showed a monophyletic introduction, given that the three samples are grouped into a single arm.

This study did not identify any major mutations of transmitted drug resistance in the samples from naive pregnant women. In a study carried out in Goiânia^26^ and in another in Rio de Janeiro,^18^ transmitted drug resistance among naive pregnant women was identified in 9.3% and 17.2% of them, respectively. Studies on HIV-1 infected individuals without antiretroviral exposure have reported prevalences of transmitted drug resistance ranging from 4.2% to 18.2%.^12^^,^^13^^,^^16^^,^^17^^,^^25^^,^^27^ Ferreira et al.^22^ found that the prevalence of transmitted drug resistance in São Paulo and Campinas was 7.6% and that 76.0% of these cases carried mutations for resistance to NNRTIs.

Among the cARVT-exposed pregnant women, major resistance mutations were present in 35.0%. However, this may have been an overestimation, due to the small sample size in this study. The most frequent mutation was M184V, which caused resistance to lamivudine and intermediate resistance to abacavir and didanosine, along with increased susceptibility to zidovudine, stavudine and tenofovir. Three pregnant women whose HIV-1 strains presented this mutation had already used cARVT containing lamivudine. Other studies on patients who had already been treated with several antiretroviral regimens also observed higher frequency of the M184V mutation, in up to 88.0% of the individuals evaluated.^14^^,^^17^^,^^24^

It was expected to find drug-resistant mutations among women who had had a diagnosis of HIV infection for a longer time, had had a previous event of opportunistic infection and presented a worse clinical and immunological stage of the disease. However, cARVT-exposed pregnant women who had had a diagnosis of HIV infection for up to five years did not have any mutations in the reverse transcriptase segment and only had accessory mutations present in protease segment. On the other hand, among the five pregnant women who had had a diagnosis of HIV infection for 10 years or more, only one did not have any major drug-resistant mutations (data not shown).

Although this study was conducted using samples collected in 2010 and 2011, this is the first to address the diversity and drug resistance of mutations in HIV-infected pregnant women in Ribeirão Preto.

## CONCLUSION

HIV-1 subtype B predominated among the pregnant women in Ribeirão Preto, state of São Paulo. This epidemiological pattern resembled what has been described in other regions of Brazil, except for the southern region. The results obtained in this study showed that no major mutations conferring drug resistance were found in the naive pregnant women, but that major mutations were found in 35.0% of the cARVT-exposed pregnant women.
